# *NME8* rs2718058 polymorphism with Alzheimer's disease risk: a replication and meta-analysis

**DOI:** 10.18632/oncotarget.9086

**Published:** 2016-04-28

**Authors:** Shu-Lei Liu, Xue-Chun Wang, Meng-Shan Tan, Hui-Fu Wang, Wei Zhang, Zi-Xuan Wang, Jin-Tai Yu, Lan Tan

**Affiliations:** ^1^ Department of Neurology, Qingdao Municipal Hospital, School of Medicine, Qingdao University, Qingdao, PR China; ^2^ Department of Radiology, Qingdao Municipal Hospital, School of Medicine, Qingdao University, Qingdao, PR China

**Keywords:** NME8, Alzheimer's disease, association study, polymorphism, meta-analysis

## Abstract

Recently, a large meta-analysis of five genome wide association studies (GWAS) has identified that a novel single nucleotide polymorphism (SNP) rs2718058, adjacent to gene *NME8* on chromosome 7p14.1, was associated with late-onset Alzheimer's disease (LOAD) in Caucasians. However, the effect of rs2718058 on other populations remains unclear. In order to explore the relationship between rs2718058 and LOAD risk in a North Han Chinese population, we recruited 984 LOAD cases and 1354 healthy controls that matched for sex and age in this study. The results showed no significant differences in the genotypic or allelic distributions of rs2718058 polymorphism between LOAD cases and healthy controls, even though after stratification for *APOE* ε4 status and statistical adjustment for age, gender and *APOE* ε4 status (*p* > 0.05). However, a meta-analysis conducted in a sample of 82513 individuals confirmed a significant association between SNP rs2718058 and LOAD risk (OR = 1.08, 95%CI = 1.05–1.11) in the whole population. But there was still no positive results in Chinese subgroup (OR = 1.05, 95%CI = 0.93–1.17). In conclusion, the rs2718058 near gene *NME8* on chromosome 7p14.1 might not play a major role in the genetic predisposition to LOAD in the North Han Chinese.

## INTRODUCTION

Alzheimer's disease (AD) is the leading cause of dementia characterized by memory loss and other cognitive impairment in adults, with over 35 million people suffering from it throughout the world [1]. In recent years, great breakthrough has been made in exploration of the molecular genetics of AD. Three genes are identified to be associated with early-onset AD (EOAD): the amyloid-β precursor protein gene (*APP*), the presenilin 1 gene (*PSEN1*) and the presenilin 2 (*PSEN2*) gene [2]. Together, the mutations of above genes are responsible for 30 to 50% of EOAD cases, and about 0.5% of AD. By far, only the ε4 allele of the apolipoprotein E (*APOE*) gene has been identified to relate with the risk of the more common LOAD [2]. However, the inheritance of the *APOE* ε4 allele only represents a minority of the underlying genetic effects, with about 50% of LOAD patients not carrying it [3].

Besides the *APOE* polymorphism, additional 11 loci have been identified in a meta-analysis of these large LOAD consortium data sets, including *CASS4*, *CELF1*, *NME8*, *DSG2*, *FERMT2* and among others [4]. Herein, a new discrete locus (rs2718058) adjacent to *NME8* on chromosome 7p14.1 was identified as a protective factor for AD in the two stages of the meta-analysis. The *NME8* (encoding NME/NM23 family member 8), owning to its role in the cytoskeletal function, axonal transport and antioxidant action, has been defined as a functional candidate gene for LOAD [4]. What's more, the variation in *NME8* could act as an eQTL (expression quantitative trait loci) for other gene(s) whose expression is directly relative to AD risk [5]. Consistent with this, another important study also reveals that the *NME8* (rs2718058) could delay the cognitive decline and play a preventive role in the development of AD [6]. The significant association of the SNP rs2718058 near *NME8* on chromosome 7p14.1 and LOAD in the above studies was reported in the Caucasian population. As variations and frequencies of gene might be different in various ethnic groups, a replication study is required to confirming the potential effects of rs2718058 in non-Caucasian populations including Asians. Up till now, SNP rs2718058 on chromosome 7p14.1 has not been examined in a North Han Chinese population. Therefore, in order to affirm this question, a case-control study was conducted to assess the association between SNP rs2718058 near *NME8* on chromosome 7p14.1 and LOAD in a Northern Han Chinese.

## RESULTS

We studied 2338 ethnic Northern Han Chinese subjects including a total of 984 subjects with probable LOAD and 1354 healthy control subjects. The demographic and clinical characteristics of LOAD and control subjects are summarized in Table 1. No statistically significant differences were observed for age (age at onset for LOAD and age at examination for controls) and gender (*P* > 0.05) between LOAD case group and control group. The MMSE scores were significantly less in AD patients than in controls (*P* < 0.001). As expected, the *APOE* ε4 allele frequency was also significantly different between AD patients and controls (*P* < 0.001, OR = 2.422, 95%CI = 1.970~2.977).

**Table 1:** The characteristics of the study population

	AD (*n* = 984)	Controls (*n* = 1354)	*P*	OR (95% CI)
Age, years; mean ± SD	75.15 ± 6.08	75.50 ± 6.49	0.186*	
Gender, n (%)			0.068	
Male	406 (41.3)	610 (45.1)		
Female	578 (58.7)	744 (54.9)		
MMSE score, mean ± SD	11.94 ± 6.20	28.49 ± 1.09	< 0.001	
*APOE* ε4 status, n (%)			< 0.001	
*APOE* ε4 (+)	280 (28.5)	191 (14.1)		2.422 (1.970~2.977)
*APOE* ε4 (−)	704 (71.5)	1163 (85.9)		

Abbreviations: AD, Alzheimer's disease; HC, healthy controls; MMSE, Mini-Mental State Examination; ApoE; apolipoprotein E; SD, standard deviation.

* *P* value was calculated with the age at onset for late-onset AD and age at examination for control. Differences in the characteristics of age and MMSE score between the two groups were examined using Student's *t* test. Differences in gender and ApoE-ε4 frequency between AD patients and HC were assessed using the Pearson χ2 test.

The genotype and allele distributions of rs2718058 in the cases and controls in the total sample and after stratification for *APOE* ε4 allele are presented in Table 2. Distributions of the rs2718058 genotypes in controls was in the Hardy-Weinberg equilibrium (HWE) (*P* > 0.05) and that in case group was not in the Hardy-Weinberg equilibrium (*P* = 0.02361). All these were in consistent with the HWE except in AD group. Careful examination of the genotyping results did not reveal any genotyping errors. This might be associated with the sample size, the geographical distribution of the population, random genetic drift, or other uncertain factors. The frequency of the minor allele G was higher in LOAD compared to controls (22.9% versus 21.3%). However, there was no significant difference between LOAD patients and controls (OR = 1.072, 95% CI = 0.932~1.234, *P* = 0.331). Similarly, the genotypes were not significantly different from LOAD patients and controls (*P* = 0.181). What's more, when these data were stratified by the *APOE ε4* status, there were still no evident differences in the genotypicor allelic distributions between AD cases and controls (Table 2). Furthermore, the results of the multivariate logistic regression with adjustment for age, gender, and the carriage of at least one *APOE* ε4 allele also failed to reveal any significant difference between LOAD and controls (Dominant: OR = 1.091, 95% CI = 0.919~1.295, *P* = 0.319; Recessive: OR = 1.341, 95% CI = 0.927~1.941, *P* = 0.119; and Additive: OR = 1.106, 95%CI = 0.961~1.273, *P* = 0.160).

**Table 2:** Genotype frequencies of the SNP rs2718058 in total subjects stratified by ApoE ε4 status

rs2718058	*N*	Genotypes (*n* %)				Alles (*n* %)			
		A/A	A/G	G/G	*P*	A	G	*P*	OR (95% CI)
Total samples									
AD	984	604 (61.4)	318 (32.3)	62 (6.3)	0.181	1526 (77.5)	442 (22.5)	0.331	1.072 0.932~1.234
Control	1354	840 (62.0)	452 (33.4)	62 (4.6)		2132 (78.4)	576 (21.6)		
ApoEε4 carriers									
AD	280	190 (67.9)	72 (25.7)	18 (6.4)	0.520	452 (80.7)	108 (19.3)	0.177	0.804 (0.585~1.104)
Control	191	120 (62.8)	56 (29.3)	15 (7.9)		296 (77.1)	88 (22.9)		
ApoEε4non-carriers									
AD	704	414 (58.8)	246 (35.0)	44 (6.2)	0.061	1074 (76.3)	334 (23.7)	0.052	1.170 0.999~1.371
Control	1163	720 62.0)	396 (34.0)	47 (4.0)		1836 (79)	488 (21)		

ApoEε4 carrier: Subjects who contain 1 or 2 ε4 alleles;

ApoEε4 non-carrier: Subjects who do not contain ε4allele.

**Figure 1:**
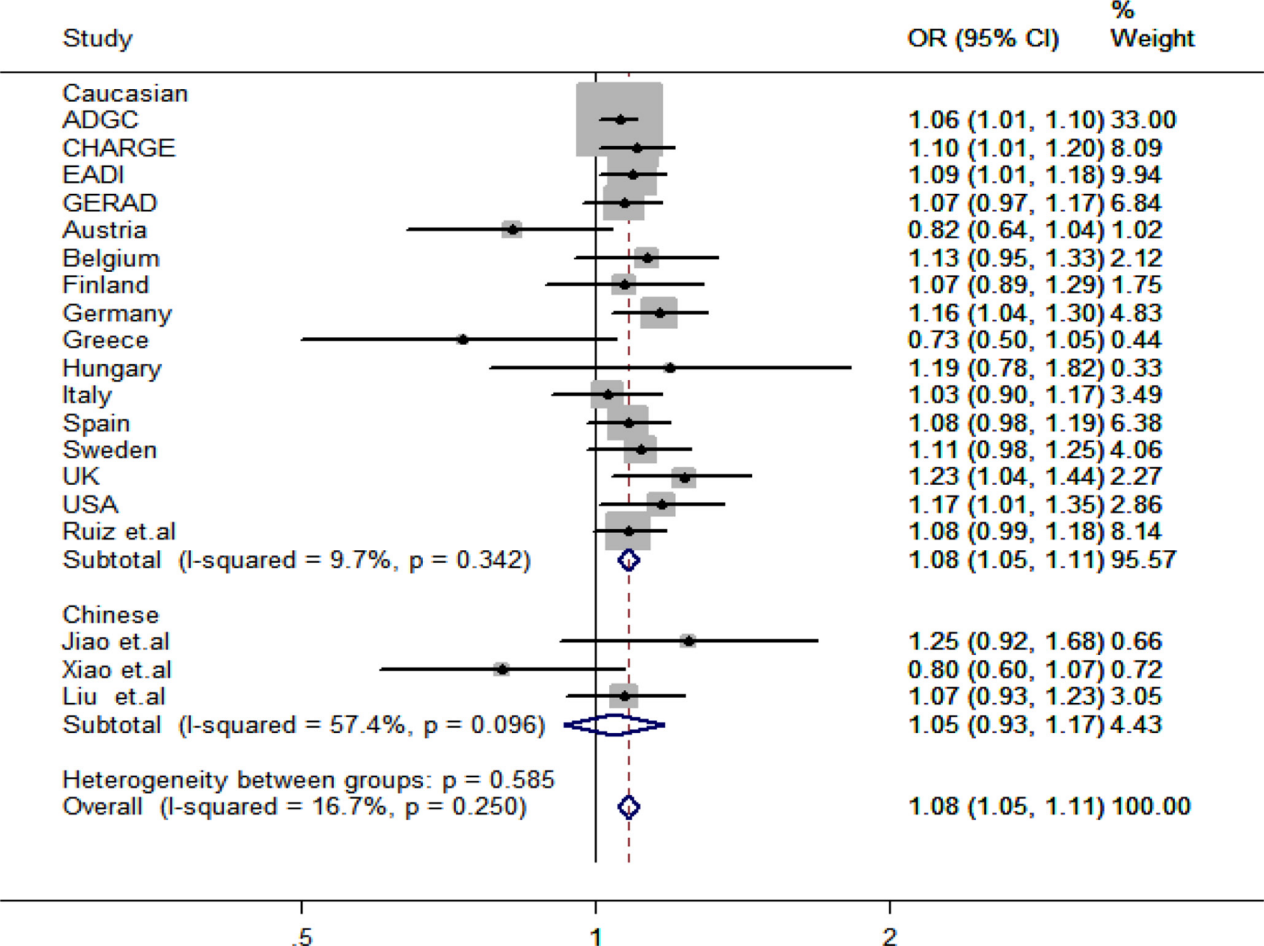
Forest plot for rs2718058 in LOAD and healthy controls in 82513 individuals

Finally, we conducted a meta-analysis which combined the results from Caucasian population, South Han Chinese population and North Han Chinese population together on the association of rs2718058 and LOAD in 82513 individuals and found an increased risk in LOAD (OR = 1.08, 95%CI = 1.05~1.11) (Figure 1) without evident analysis heterogeneity (*I*^2^ = 16.7%). However, to our disappointment, we still failed to find an effect of rs2718058 on LOAD in Chinese population (OR = 1.05, 95%CI = 0.93~1.17) with evident analysis heterogeneity (*I*^2^ = 57.4%).

## DISCUSSION

Using a large cohort of 984 LOAD cases and 1354 controls, we did not replicate in North Han Chinese the association of the rs2718058 SNP on chromosome 7p14.1 that is reported as associated with LOAD in Caucasians. In accordance with our results, Xiao et. al's study [7] and Jiao et al's. study [8], both conducted in south Han Chinese cohorts, also failed to detect an association between SNP rs2718058 and LOAD risk.

**Figure 2:**
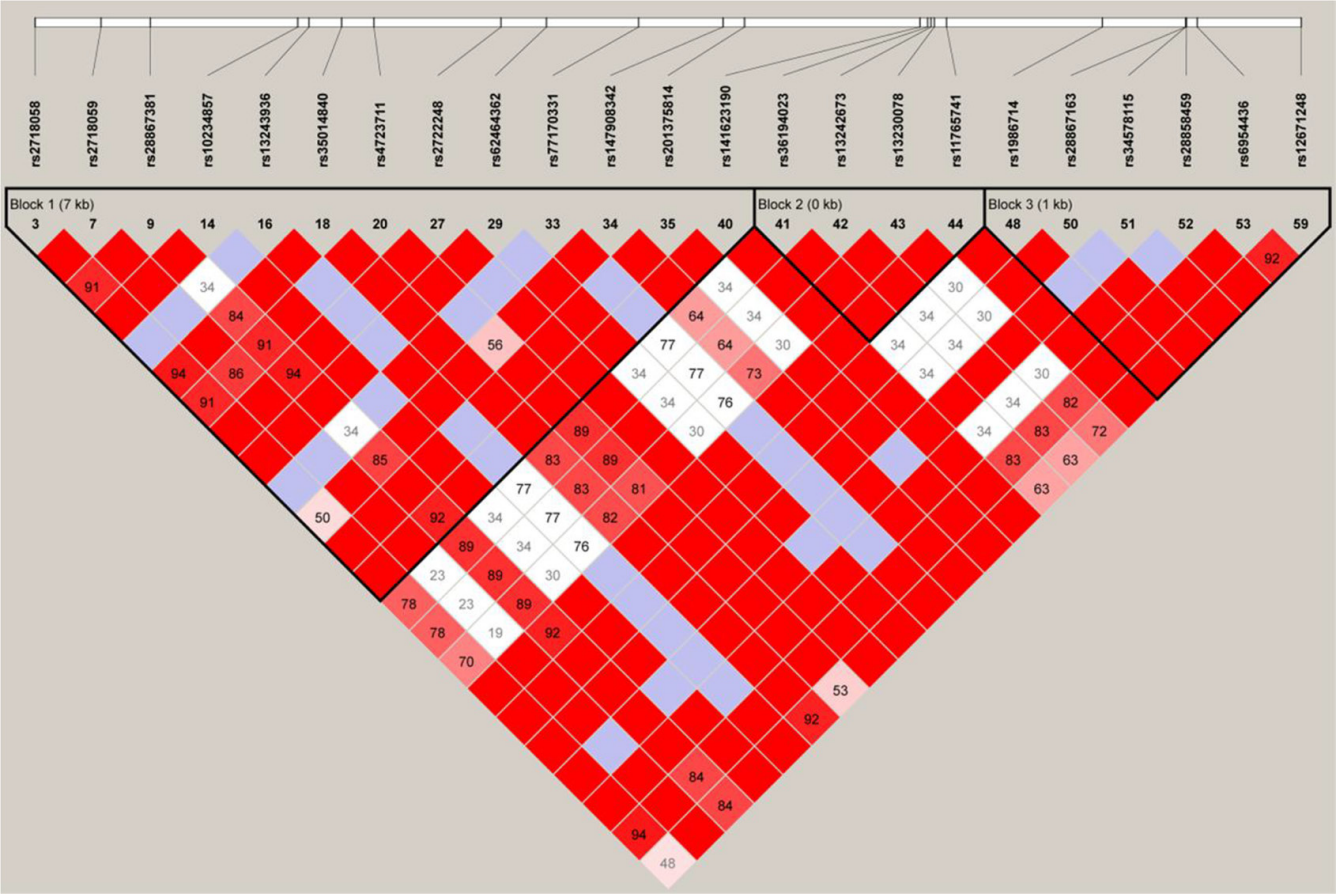
LD structure of all the SNPs in LD with rs277180058 in the European-descendent population The LD structure around rs2718058 was determined using Haploview software. The standard LD color scheme was used (D'/LOD) with white to red colors representing the increasing strength of LD.

**Figure 3:**
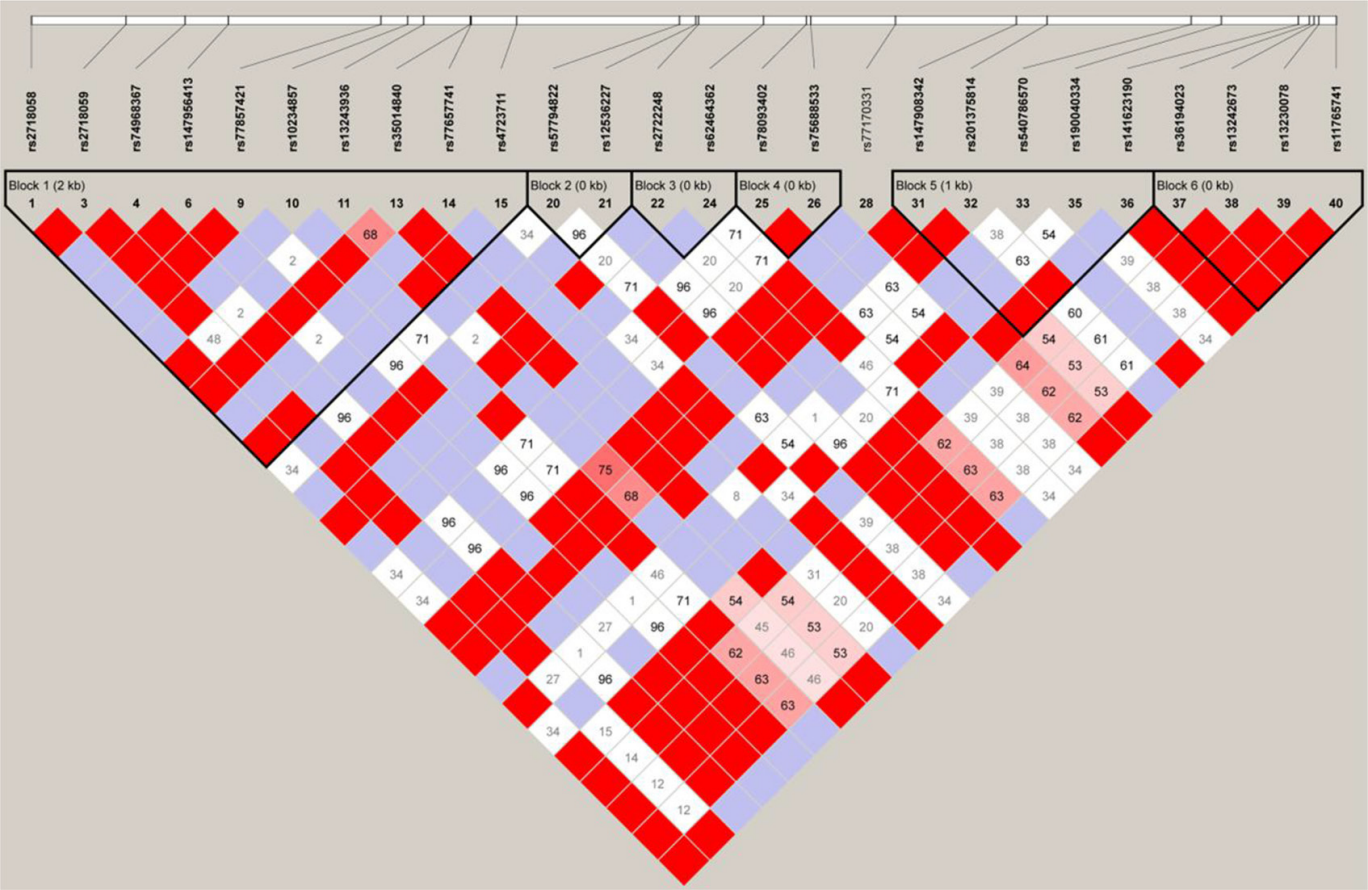
LD structure of all the SNPs in LD with rs27118058 in the Han Chinese population The LD structure around rs2718058 was determined using Haploview software. The standard LD color scheme was used (D'/LOD) with white to red colors representing the increasing strength of LD.

Although we failed to obtain analogous relation in our population, this does not necessarily invalidate previous data. Several factors might account for a failure to replicate findings. Firstly, one vital factor is the genetic heterogeneity in different ethnic populations, containing differences of minor allele frequency (MAF) and the underlying complicated genetic architecture. Our study indicated that the MAF of rs2718058 was different from that in Caucasians from the SNP database, with the lower MAF (G allele) in the Han Chinese (22% vs 36%). Moreover, we utilized two populations (CEU and CHB) from Hapmap database to investigate linkage disequilibrium (LD) structure of all the SNPs in LD with rs2718058. The LD structure present in European descendant were different from that present in the Han Chinese population. Interestingly, in CEU LD analysis, we observe that rs2718058 was tightly linked to 12 important SNPs (rs2718059, rs28867381, rs10234857, rs13243936, rs35014840, rs4723711, rs2722248, rs62464362, rs77170331, rs147908342, rs201375814, rs141523190) within a same LD block in *NME8* gene (Figure 2). Among the 12 SNPs, the rs4723711 (1.778 × 10^−8^) and rs2722248 (3.433 × 10^−5^) are strongly associated with LOAD respectively. However, in CHB LD analysis, the rs2718058 was not in LD with these SNPs in *NME8* gene (Figure 3). Secondly, the effects of the genetic variants confirmed by GWAS and the Liu et al. [6] might be population-specific, in view of the unknown specific gene–gene or gene–environment interactions. A brain structural mechanism for such population-specific genetic effects probably makes effects of rs2718058 on AD diverse [9]. Thirdly, the variable sample size is another common factor leading to the different results. There is some trend for association in *APOE ε4* non-carriers, with a *p*-value of 0.06 for genotype comparisons and 0.05 for allele comparisons. Although our sample size had a more than 90% power to detect a modest risk factor at a significance level (alpha) of 0.05 based on the minor allele (G) frequency in our study, the power decreased with stratification on *APOE ε4* status. It is possible that increasing the sample size could lead to some significant results. The power to detect association in the *APOE ε4* non-carriers subgroup is 65.2%. The discrepancy in *APOE* non-carriers may possibly due to a limited number of cases and controls of *APOE* non-carriers in such a small sub-cohort. Thus it should be validated in a larger cohort in the future. Lastly, except for the specific population and particular genetic background or environment, the variations of the sample clinical characteristics, for example the onset age, interaction between several other unknown neuropsychiatric changes as well as experiment methods and statistical analyses may engender statistical deviation [10]. Furthermore, in order to avert above mentioned possible complex reasons and further investigate these associations, a meta-analysis that combined the results from Caucasian population, south Han Chinese population and North Han Chinese population together was conducted. Although a strong relation between SNP rs2718058 with LOAD risk was detected out in the entire population, we still failed to confirm a positive association in the Chinese subgroup. Hence, this association between SNP rs2718058 with LOAD might mainly consist in Caucasians cohort.

In contrast with our data, the large meta-analysis of GWAS identified rs2718058 as a protector for LOAD, which might participate in the cytoskeletal function, axonal transport and antioxidant action [4]. Similarly, Liu et al also found that the functional genetic variant adjacent to *NME8* had a preventive effect on the brain neurodegeneration and could delay cognitive decline. Moreover, rs2718058 variants significantly associate with several AD related endophenotypes including the elevated tau levels in CSF, the hippocampus atrophy, occipital gyrus atrophy, lateral ventricle hypometabolism throughout the AD physiopathological process [6].

In summary, our study suggests that the rs2718058 polymorphism may not act as a major factor in progression of LOAD in the North Han Chinese population. It is probably that the effect of rs2718058 SNP on AD risk is specific to some particular ethnic populations or that the effect is not large enough to be detected reliably by a sample of our size. In view of that this is the first study investigating the possible contribution of rs2718058 polymorphism to LOAD in the north Han; therefore, present results require confirmation in further and larger studies in north Han Chinese as well as in other ethnic groups.

## MATERIALS AND METHODS

### Subjects

Our study is consisted of 984 sporadic LOAD patients (age at onset ≥ 65 years) and 1354 healthy individuals matched for gender and age. All above LOAD patients and control subjects were uncorrelated Han Chinese residents from Shandong Province. The patients were conscribed from the Department of Neurology of the Qingdao Municipal Hospital, and several other 3A-level hospitals; they were subjected to neuropsychological examination, structural neuroimaging consisting of brain computed tomography and/or magnetic resonance imaging. A consistent clinical diagnosis of probable AD was established by at least two neurologists in accordance with the criteria of National Institute of Neurological and Communicative Disorders and Stroke and the Alzheimer's disease and Related Disorders Association (NINCDS–ADRDA) [11]. All AD patients were sporadic and none of their first-degree relatives had dementia in their family history. Age at onset and family history were determined from caregivers. The controls were matched with the patients in terms of sex, age and confirmed to be free of mental illness after undergoing a health examination, including medical history, general examinations, laboratory examinations and Mini Mental State Examination (MMSE) score. Demographic details of the sample set are revealed in Table 1. An informed consent to participate in this study was obtained from all subjects or from a guardian, and the protocol of this study was approved by the Ethical Committee of Qingdao Municipal Hospital [12].

### Genotype analysis

Human genomic DNA was extracted from peripheral blood leukocytes of AD patients and healthy individuals using the Wizard Genomic DNA Purification Kit (Cat. #A1125, Promega, USA) following the manufacturer's protocol. The *NME8* (rs2718058) polymorphismsis genotyped with the SNPscan technique using SNPscan^™^ kit (Genesky Biotechnologies Inc., Shanghai, China) to design and determine the genotypes [13]. This is a high-throughput and cost-saving SNP genotyping method, which was based on double ligation and multiplex fluorescence PCR [14, 15]. 5% of the high DNA quality samples were randomly subjected to repeated analyses to guarantee the genotyping qualities. The average genotype call rate for all markers was more than 96%. *APOE* genotypes were determined as the method described by Donohoe et al. [16].

### Statistical analysis

The statistical analyses were performed by SPSS 16.0 software. The Hardy–Weinberg equilibrium (HWE) was tested using χ2 test. Genotype and allele frequencies were calculated by counting. The characteristics for AD patients and control subjects were assessed by the Student-*t* test or the χ2 test. Differences in allele and genotype frequencies of the two groups were assessed using the Pearson χ2 test or Fisher's exact test. Multivariate logistic regression analyses, adjusting for gender, *APOE* ε4 status and age at onset or age at examination, were used to estimate odds ratios (ORs) with 95% confidence intervals (CIs) for assessing genotypic and allelic associations with AD under various genetic models that were defined as 1 (aa+Aa) versus 0 (AA) for dominant,1 (aa) versus 0 (AA+Aa) for recessive, and 0 (AA) versus 1 (Aa) versus 2 (aa) for additive (A: major allele; a: minor allele). Estimation of the statistical power was performed with the STPLAN 4.3 software. The level of significance for all statistical tests was defined as *P* < 0.05.

Moreover, we combined our data with the results from large meta-analysis of 74046 individuals [4] and other studies [7, 8, 17] on *NME8* (rs2718058) and LOAD by fixed-effects inverse variance-weighted methods. Meanwhile, we generated *I*^2^ estimates with evaluate the possible effect of study heterogeneity on the results. We used Stata V.12.0 to perform all the meta-analyses.
